# Canine Vector-Borne Diseases (CVBDs) in Liguria, North-West Italy: A Retrospective Study over an 11-Year Period (2013–2023)

**DOI:** 10.3390/ani14233539

**Published:** 2024-12-07

**Authors:** Sara A. Chiarlone, Aitor Garcia-Vozmediano, Valentina Virginia Ebani, Nicola Pussini, Monica Dellepiane, Lisa Guardone, Elisabetta Razzuoli

**Affiliations:** 1 S.S. Ponente Ligure, Istituto Zooprofilattico Sperimentale del Piemonte, Liguria e Valle d’Aosta, Via Martini 6, 17056 Savona, Italy; sara.chiarlone@izsplv.it (S.A.C.); nicola.pussini@izsplv.it (N.P.); dellepiane.vet@gmail.com (M.D.); 2 S.S. Epidemiologia Sicurezza Alimentare, Istituto Zooprofilattico Sperimentale del Piemonte, Liguria e Valle d’Aosta, Via Bologna 220 c/o Lanificio di Torino, 10154 Torino, Italy; aitor.garciavozmediano@izsplv.it; 3 Department of Veterinary Sciences, University of Pisa, Viale delle Piagge 2, 56124 Pisa, Italy; valentina.virginia.ebani@unipi.it; 4 Centre for Climate Change Impact, University of Pisa, 56124 Pisa, Italy; 5 S.S. Genova e Portualità Marittima, Istituto Zooprofilattico Sperimentale del Piemonte, Liguria e Valle d’Aosta, Piazza Borgo Pila 39-24, 16129 Genova, Italy; elisabetta.razzuoli@izsplv.it

**Keywords:** *Anaplasma* spp., *Borrelia burgdorferi* sensu lato, *Dirofilaria immitis*, dogs, *Ehrlichia* spp., *Rickettsia* spp., seroprevalence

## Abstract

Canine vector-borne diseases (CVBDs) pose a global threat to both canine and public health. This study evaluates the prevalence of *Anaplasma* spp., *Ehrlichia* spp., *Borrelia burgdorferi* sensu lato (s.l.), *Rickettsia conorii*, and *Dirofilaria immitis* in domestic dogs in Liguria, north-west Italy, a region where data were lacking. From 2013 to 2023, 8584 blood samples from shelter (74%) and owned dogs (26%) were submitted to the *Istituto Zooprofilattico Sperimentale of Piemonte, Liguria and Valle d’Aosta* (IZSPLV) for serological testing (indirect immunofluorescence and/or rapid immunochromatographic tests). Overall, 18.8% (95% CI: 18.0–19.7) of the dogs tested positive for at least one pathogen, with positivity against *R. conorii* antigen being the most frequently recorded (24.4%, 95% CI: 23.3–25.5). Lower prevalence levels were observed for *Anaplasma* spp. (1.82%, 95% CI: 1.47–2.23), *Ehrlichia* spp., (1.25%, 95% CI: 0.97–1.60), *B. burgdorferi* s.l. (0.22%, 95% CI: 0.11–0.39), and *D. immitis* (0.84%, 95% CI: 0.65–1.06). Positive cases for all pathogens increased over time, with prevalence differing significantly between owned and shelter dogs. Topographical factors, land use, and monthly relative humidity appeared to influence the positivity in the dogs. These results update the epidemiology of the investigated CVBDs in Liguria, indicating a widespread exposure to *Rickettsia* spp. among local dogs.

## Simple Summary

Dogs can be affected by diseases transmitted by arthropod vectors, some of which can also be transmitted to humans. The aim of this study was to present the results of diagnostic tests for selected pathogens (*Anaplasma* spp., *Ehrlichia* spp., *Borrelia burgdorferi* sensu lato (s.l.), *Rickettsia conorii*, and *Dirofilaria immitis*) conducted by a public veterinary diagnostic laboratory (*Istituto Zooprofilattico Sperimentale of Piemonte, Liguria and Valle d’Aosta, IZSPLV*) in Liguria, north-west Italy, over an 11-year period (2013–2023). Overall, 18.8% of the dogs tested positive for at least one pathogen. In detail, most positive dogs showed exposure to *R. conorii*, followed by *Anaplasma* spp., *Ehrlichia* sp., *D. immitis*, and *B. burgdorferi* s.l. This work contributes to the description of the epidemiology of the selected pathogens in Liguria, showing a widespread exposure of dogs to *Rickettsia* spp., while the other pathogens appear to be less frequent compared to other areas of Italy.

## 1. Introduction

The term canine vector-borne diseases (CVBDs) encompasses a range of infectious diseases transmitted by arthropod vectors [1,2]. In addition to representing a significant threat to canine health on a global scale [3,4,5,6], several of these diseases are zoonotic, thus being a hazard to public health [1,2,7,8]. The risks posed by CVBDs to human and animal health are increasingly recognised and influenced by factors such as global warming, human and canine population growth and the expansion of international mobility and trade [1,2,9].

Among the most significant canine vector-borne pathogens (CVBPs), the tick-borne bacteria of the genera *Anaplasma*, *Borrelia*, *Ehrlichia*, and *Rickettsia*, along with the mosquito-transmitted heartworm, *Dirofilaria immitis*, are the most commonly found in dogs from Europe [5,10,11]. *Anaplasma phagocytophilum* and *Anaplasma platys* are responsible for granulocytic anaplasmosis and canine infectious cyclic thrombocytopenia, respectively [12]. *Anaplasma phagocytophilum* is primarily transmitted by ticks of the genus *Ixodes*, particularly *Ixodes ricinus* in Europe, although it has also been documented in ticks of the genus *Rhipicephalus* and *Hyalomma* [13]. Anaplasmosis in dogs has been recorded worldwide [3,14,15,16]. In Italy, several studies have reported the presence of members of the Anaplasmataceae family in dogs; however, the results are variable, depending on the diagnostic test used, the geographical context and the type of dog population included [12,17]. The zoonotic potential of *A. phagocytophilum* is well-known [3,14], while for *A. platys* it is still unclear [18,19].

*Ehrlichia canis* is the causative agent of canine monocytotropic ehrlichiosis (CME), a severe and potentially fatal immunosuppressive disease occurring worldwide, particularly in tropical and subtropical regions [20]. It is transmitted globally by the brown dog tick, *Rhipicephalus sanguineus* sensu lato (s.l.) [20]. A zoonotic role has been supposed after *E. canis* was found in clinical samples from humans with clinical signs similar to those of CME [21]. In addition, *Ε. ewingii* is responsible for granulocytic ehrlichiosis in dogs [22], although in Europe only *E. canis* has been identified so far [23]. This species is common in Italy, where it was reported with variable seroprevalence values [12,13,17,24,25,26,27].

The genus *Rickettsia* includes several species able to cause disease in animals and humans, classified in the spotted fever group (SFG) and typhus group (TG). Within the SFG, *R. conorii* is the most commonly described, and it is responsible for the disease called Mediterranean spotted fever. Its main vector is *R. sanguineus* s.l. Other SFG rickettsiae have been found in animals and arthropod specimens in Europe and in Italy [17]. These include *R. helvetica* and *R. monacensis* (mainly transmitted by *I. ricinus)*, *R. massiliae* (mainly transmitted by ticks belonging to *R. sanguineus* s.l.), *R. aeschlimannii* (mainly transmitted by *Hyalomma* spp.), *R. slovaca*, and *R. raoultii* (mainly transmitted by *Dermacentor marginatus* and *D. reticulatus* ticks) [7,28,29,30,31].

*Borrelia burgdorferi* sensu lato (s.l.) is a zoonotic tick-borne pathogen (TBP), responsible for Lyme borreliosis and mainly transmitted by *I. ricinus* [32]. This pathogen has been reported in dogs from numerous countries across the European continent. A recent comprehensive study provides an overview of the European canine seroprevalence of this species, revealing a significant inter-country variability, ranging from 0.2% to 10.2%. The highest values were concentrated in northern and eastern countries [32]. Its presence in dogs is also known in Italy [4,33,34].

Besides TBDs, another relevant CVBD is dirofilariosis, a disease transmitted by mosquitoes of the genus *Culex* and *Aedes* [35,36,37]. Dogs can be infected by several species of filariae, but *Dirofilaria immitis*, the aetiological agent of canine heartworm disease, is the most pathogenic species [38]. This species is also responsible for rare zoonotic infections, although most cases are caused by *D. repens* [39]. In recent decades, canine filarial infections have spread in Italy [37,40,41] and in Europe, with cases now emerging in areas previously considered free of the parasite, or for which the epidemiological status had not been documented yet [42,43,44,45,46,47].

Despite the widely acknowledged significance of CVBDs and the imperative for control measures to safeguard the health of both dogs and humans, the epidemiology and public health implications of CVBDs in Liguria, northwestern Italy, remain underexplored. To address this knowledge gap, this cross-sectional retrospective study investigates the prevalence of selected CVBPs (*Anaplasma* spp., *Ehrlichia* spp., *Rickettsia conorii, B. burgdorferi* s.l., and *D. immitis*) in Ligurian dogs over an 11-year period (2013–2023), by analysing diagnostic data from a public veterinary diagnostic institution (*Istituto Zooprofilattico Sperimentale of Piemonte, Liguria and Valle d’Aosta*—IZSPLV), along with environmental and topographical data.

## 2. Materials and Methods

### 2.1. Ethical Statement

The retrospective study only includes data obtained from the IZSPLV records, from the regular checks carried out by the Public Veterinary Service in kennels, in agreement with Italian legislation, and routine diagnostics. No sampling or analysis were carried out specifically for this work and no experimental animals were involved.

### 2.2. Serum Sampling and Analysis

All serological analyses were conducted within the laboratories of Savona section of IZSPLV, where all samples from the Liguria region are processed for CVBPs. Upon arrival, each sample was assigned a unique code, which was linked to the municipality of origin, the microchip number, and the type of environment (private house/kennel). The samples were then stored at +4 °C and analysed within 3 days. Shelter dogs were examined upon entrance to the kennel (Savona, Genova, and La Spezia) and/or as part of an annual preventive screening (Imperia), while owned dogs were tested mainly as a part of regular preventive screening, in the case of adoption from other regions, or in case of symptoms being discovered.

#### 2.2.1. Indirect Immunofluorescence Assay (IFA) for *Rickettsia conorii*

Serum samples from dogs were tested for the presence of circulating IgG antibodies against *R. conorii* antigens. The test was performed on IFA slides containing fixed cells infected with *R. conorii* (FLUO RICKETTSIA conori, Agrolabo Spa, Scarmagno (TO), Italy). Briefly, the screening phase involved the dilution of sera at a 1:40 ratio in phosphate-buffered saline (PBS, pH 7.2) and subsequent incubation of the diluted sera on slide wells for 30 min at 37 °C in a humidified chamber. Canine positive and negative controls certified by the National Reference Centre for *Anaplasma, Babesia, Rickettsia*, and *Theileria*—CRABART (*Istituto Zooprofilattico Sperimentale della Sicilia*)—were included on each slide. The slides were then rinsed twice in PBS and once in distilled water, after which they were air-dried. Subsequently, fluorescein isothiocyanate–conjugated (FITC) anti-canine IgG, supplied by CRABART as ready-to-use, was added to each well. The slides were then incubated in a humidified chamber at 37 °C for 30 min, washed and dried as previously described, and examined by fluorescence microscopy. Afterwards, samples showing fluorescence at the 1:40 dilution were tested at two-fold higher dilutions. Scores from 1 to 4 were assigned to the intensity of specific fluorescence and the antibody titre was defined as a major dilution with a score of 2. The titre 1:80 was considered as the cut-off value, as suggested by the manufacturer.

#### 2.2.2. Serological Analysis for *Anaplasma* spp., *Ehrlichia* spp., *Borrelia burgdorferi* s.l., and *Dirofilaria immitis*

Canine serum, plasma, or whole blood was employed to search for circulating antigens of *Dirofilaria immitis* and for antibodies against specific peptides of *A. phagocytophilum*, *A. platys*, *E. canis*, *E. ewingii*, and *B. burgdorferi* s.l. using a rapid immunochromatographic test (SNAP^®^ 4Dx^®^ Plus Test IDEXX Laboratories, Westbrook, ME, USA), when testing for all the aforementioned pathogens was required. A rapid immunochromatographic test targeting only the *D. immitis* antigen (Heartworm IC, Agrolabo Spa, Scarmagno (TO), Italy) was used when testing for this pathogen was exclusively requested. The tests were performed according to the manufacturer’s instructions.

### 2.3. Data Management and Processing

Only dogs for which data on the microchip and municipality was available were included in the study. Prior to data analysis, the dataset was subjected to data cleaning and processing procedures to ensure appropriate quality of the data. The main dataset was split into different subsets according to the laboratory tests conducted. For each subset, records were checked for duplicates based on the animal ID, the date of testing and the diagnosis. For individuals for which multiple tests were conducted over time, only one record was retained to ensure the accuracy and reliability of the prevalence estimates. This approach was adopted to prevent the over-representation of animals (i.e., some individuals had been tested more frequently), which could result in a bias in the results, and to ensure comparability by focusing on distinct individuals within the population. Consequently, records for individuals with multiple testing were selected based on the following criteria: (1) if all the records of the same individual displayed consistent positive or negative results for the same pathogen, only the first diagnosis was considered; (2) if the records of the same individual displayed positive and negative results, the first positive diagnosis was considered.

The dataset was further enriched with topographical features of the Ligurian territory at the municipal level, including the land use [48], the elevation, the type of geographical setting (coastal/inland) and the urbanisation level [49]. The land use categories used in this study were classified into four main groups: vegetation cover (comprising deciduous and conifers woodlands and mixed forests), agriculture (encompassing rural activities such as dryland cultivation and timber arboriculture), wetlands (including estuarine, lacustrine, palustrine, and riverine) and urban land (representing urban residential fabric). The total area occupied by each of these land use classes within each municipal limit was calculated and expressed as a percentage. Moreover, municipalities were classified according to their elevation (expressed in metres above the sea level, m a.s.l.) within four altitudinal bands (<250, 250–499, 500–750, and >750), as well as their urbanisation level, which was categorised into three groups (low, intermediate, and high population density).

Abiotic environmental factors such as temperature and water availability have been identified as the most significant limiting drivers influencing the transmission, spread, and distribution of VBDs [50,51]. Here, the proxy-derived temperature against latitude and the daily time series over 2013 to 2023 of minimum, maximum, and mean values of the relative humidity (RH) at 2m from the surface, with a 0.1° × 0.1° gridded resolution [52], were employed as climatic covariates to model the seroprevalence of the five study VBPs. As only data pertaining to the municipality of residence were available, gridded estimates of RH were collected and averaged at the municipal and monthly level.

### 2.4. Statistical Analysis

Data processing and analysis were conducted with Stata 17 (StataCorp. 2021, 4905 Lakeway Drive, College Station, TX 77845, USA) and R software (version 4.3.3). Firstly, an exploratory statistical analysis was conducted to evaluate the laboratory testing activity in time and space for each of the CVBPs under investigation and to describe the distribution of canine populations at the provincial level, taking into account the topographical characteristics. Individual counts and the corresponding percentages were calculated. The overall and pathogen-specific prevalence levels and the prevalence of co-exposure/infection, along with their exact binomial 95% confidence intervals (CIs), were calculated. To evaluate the differences in prevalence of co-exposure/infection between dog populations, the non-parametric Kruskal–Wallis test was employed. Moreover, temporal fluctuations of the prevalence were evaluated for each dog category and for each pathogen individually on a yearly and monthly basis. In particular, the moving annual prevalence was calculated across the entire study period, while a non-parametric loess regression was conducted to evaluate the overall seasonality.

A stepwise backward multivariate Poisson regression approach was employed to model the prevalence of each CVBP, with the models’ fit evaluated according to Akaike’s information criterion (AIC). In the initial stages of the modelling process, only those covariates displaying a significant level of up to *p* ≤ 0.20 were included in the model. Subsequently, the covariates that fulfilled this criterion were subjected to analysis and examination for potential confounding. Models that better explained the dog prevalence with the lowest AIC were considered optimal. Once the optimal model had been constructed, the adjusted effect of each covariate on the response variable was quantified and expressed as a prevalence–risk ratio (PR). To assess the geographic distribution of dog prevalence, the overall municipality-weighted PR for each pathogen was predicted for each municipality within the dataset. The predicted estimates were spatially interpolated through the inverse distance weighted (IDW) method to identify geographic distributional patterns of seroprevalence and potential areas at high risk.

## 3. Results

Between 2013 and 2023, 8584 dogs from 152 municipalities within the Liguria region underwent serological examination, targeting the five CVBP objects of the study. The sampled population was predominantly composed of shelter dogs (n = 6356; 74%) and to a lesser extent of owned dogs (n = 2228; 26%), except for in Savona province (Supplementary materials, Figure S1A). Most dogs resided in coastal areas within medium-to-moderately dense municipalities or large urban centres below 500 m a.s.l. (89.6% of the total; Supplementary materials, Table S1); only a minority of the examined dog population lived above this threshold, at elevations between 500 and 750 m a.s.l. While testing was conducted on a year-round basis, fluctuations in the number of tested animals were generally observed, with an evident downward trend throughout the year (Supplementary materials, Figure S1B). Indeed, 61.4% of the total number of tests were conducted from January to June, with 65.0% of the testing being conducted in the owned-dog population taking place during the spring months (March–May; Supplementary materials, Figure S1B).

**Figure 1:**
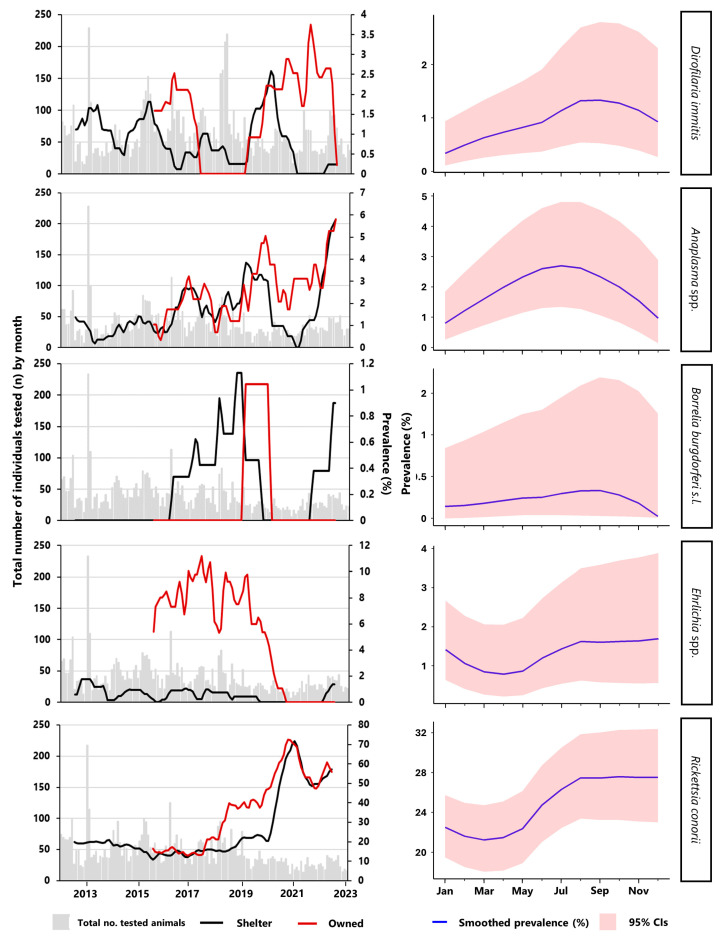
Annual moving average and seasonality of prevalence, together with the total number of individuals (shelter and owned dogs) tested in the Liguria region over the period 2013–2023 for *Dirofilaria immitis*, *Anaplasma* spp., *Borrelia burgdorferi* s.l., *Ehrlichia* spp., and *Rickettsia conorii.* Note: to accommodate the considerable heterogeneity of seroprevalence estimates identified among the various pathogens, different y-axis scales are displayed.

**Figure 2:**
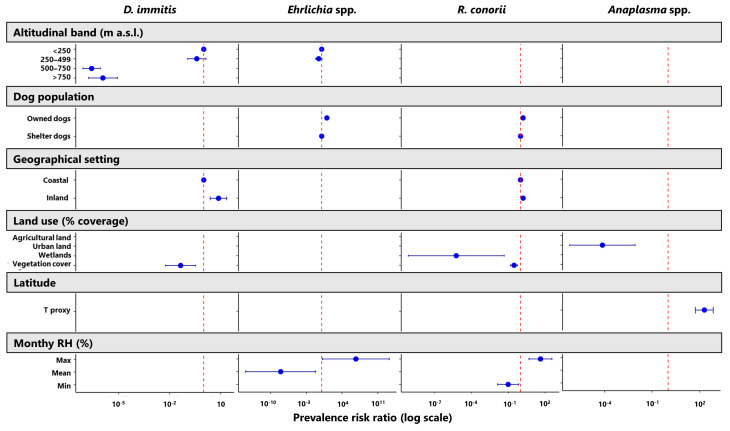
Main factors driving the prevalence of *Dirofilaria immitis*, *Anaplasma* spp., *Ehrlichia* spp., and *Rickettsia conorii* in the Liguria region between 2013 and 2023. Overall prevalence risk ratios (PRs) and 95% CIs are illustrated on a logarithmic scale (estimates can be consulted in Supplementary materials, Table S2); a deviation of point estimates and related CIs in either a negative or positive direction from PR = 1 (dashed red line) is statistically significant. For the categorical factors of the altitudinal band, dog population, and geographical setting, a PR = 1 indicates the category of reference.

Serological testing for *D. immitis* antigens and *R. conorii* antibodies were the most frequently conducted tests, with 8368 and 6024 tested dogs, respectively. No particular pattern of testing requests was generally observed over time; however, a slight increase in the number of dogs tested for *D. immitis* was observed between 2016 and 2019 (Figure 1). Overall, 18.8% (95% CI = 18.0–19.7) of the dogs tested positive for at least one of the target CVBPs (Table 1). Positivity against *R. conorii* antigen was the most frequently detected, as it was observed in 24.4% (95% CI: 23.3–25.5%) of the tested dogs (Table 1), with antibody titres ranging between >1:80 and 1:640 (Table 2). Lower prevalence values were found for the remaining CVBPs: the seroprevalence for *Anaplasma* spp. was 1.82% (95% CI: 1.47–2.23%), followed by *Ehrlichia* spp. (1.25%, 95% CI: 0.97–1.60%), and *B. burgdorferi* s.l. (0.22%, 95% CI: 0.11–0.39%), while the prevalence of *D. immitis* was 0.84% (95% CI: 0.65–1.06%) (Table 1). Differences were observed between the two study populations, with owned dogs often exhibiting higher prevalence levels, especially for *Ehrlichia* spp. (6.53% vs. 0.5%) and *R. conorii* (32.3% vs. 23.6%) (Table 1; Figure 1 and Figure 2). Despite a higher prevalence for *Anaplasma* spp. being observed in owned dogs (3.18% vs. 1.63; Table 1), exposure to *Anaplasma* spp. and *B. burgdorferi* s.l. was found to be comparable between dog populations, as was the infection prevalence of *D. immitis*. Concomitant seropositivity for two or more pathogens occurred only in 1% (95% CI: 0.8–1.22) of the Ligurian dog population examined. Of the twenty-six possible exposure/infection combinations, only nine were identified, with dual exposure being the most frequently ascertained (n = 81; 95.3%): positivity against *R. conorii* antigen in combination with *Anaplasma* spp. (n = 41; 48.2%), *Ehrlichia* spp. (n = 20; 23.5%) or *D. immitis* (n = 10; 11.8%) were the most prevalent. Interestingly, a positive result for four pathogens (*D. immitis*, *Anaplasma* spp., *Ehrlichia* spp., and *R. conorii*) was observed in a single shelter individual (Table 3). The occurrence of pathogen co-exposures was found to be statistically comparable between owned and shelter dogs (Kruskal–Wallis rank test, *p* > 0.05), although a greater number of co-exposed/infected individuals was recorded in the shelter population (n = 60/85).

In general, the number of positive individuals followed an upward trend over time and within the years studied (*p* < 0.001). The annual increases were most pronounced for *Anaplasma* spp. and *Rickettsia* spp. (Figure 1), which displayed comparable rising rates (PRadj = 1.14 and PRadj = 1.15, respectively). Similarly, we observed a noteworthy increase in the seroprevalence of *B. burgdorferi*, particularly in instances where seropositivity had been recorded (PRadj = 1.22; 95% CI = 1.01–1.49) (Figure 1), although the number of dogs exhibiting positive results was relatively limited (Table 1). Fluctuations in the prevalence across the seasons did not appear to follow any discernible pattern, with the exception of the temporal shifts observed for *D. immitis* infections. Indeed, the number of dogs that were diagnosed with dirofilariosis generally showed an increase over the seasons (PRadj = 1.28; 95% CI = 1.01–1.63), reaching a maximum peak of cases during the autumn months (1.33%; Figure 1).

**Figure 3:**
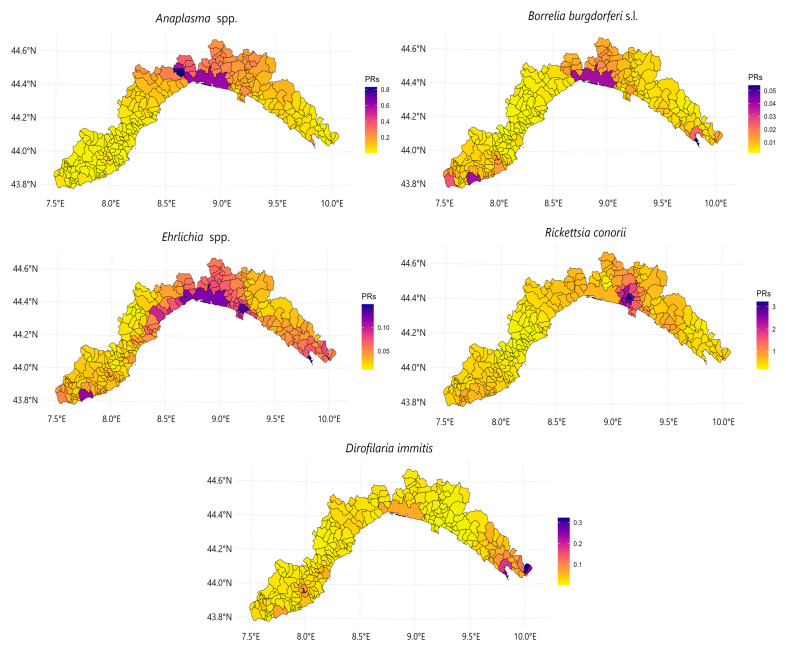
Spatial interpolation of municipality-weighted averages of prevalence risk ratios (PRs) for *Anaplasma* spp., *Borrelia burgdorferi* s.l., *Ehrlichia* spp., *Rickettsia conorii*, and *Dirofilaria immitis* across the Liguria region, northwest Italy. Note: to accommodate the considerable heterogeneity of PR estimates identified among the various pathogens, pathogen-specific scales are displayed.

The observed prevalence values also appeared to be influenced by factors related to the territorial characteristics of the region (Figure 2). Dogs residing in inland settings were more prone to testing positive against the *R. conorii* antigen and for the *D. immitis* antigen, regardless of the dog population (Supplementary materials, Table S1). In particular, the prevalence of *D. immitis* infections in dogs from inland settlements (1.35%; 95% CI = 0.73–2.25) almost doubled that observed among dogs living close to the coastline (0.8%; 95% CI = 0.58–0.99), with apparently higher positive rates being recorded in owned dogs (1.94%) than in shelter dogs (0.87%). A comparable effect was also detected for positivity against *R. conorii* antigen, even though the difference between inland and coastal areas was less pronounced (27.4% vs. 24.0%). Furthermore, a slight altitudinal trend appeared to influence *D. immitis* cases and *Ehrlichia* spp. exposure, with a tendency for these to occur below 500 m a.s.l. However, this effect was only marginally significant for *Ehrlichia* spp. seroprevalence (PR ≈ 1; Figure 2). In contrast, dogs exposed to *Anaplasma* spp. were found to be favoured by the latitude (PRadj = 194.9; 95% CI = 52.9–718.6), with the most northerly areas exhibiting a 194% increase in *Anaplasma*-positivity rates (Figure 3). With regard to land use factors, only negative associations were identified, especially for *Dirofilaria* infections and *Rickettsia* spp. exposure with vegetation coverage and wetlands areas, and *Anaplasma* spp. with urban land cover (Figure 3; Supplementary materials, Table S1). Instead, positive associations for the exposure to *Ehrlichia* spp. and *Rickettsia* spp. seemed to occur with the environmental relative humidity (Figure 2). This relation was mainly observed with the maximum monthly average of this environmental parameter, while the monthly mean and minimum values of RH exhibited a negative association for *Ehrlichia* spp. and *Rickettsia* spp. exposure, respectively.

Apart from the northern latitudinal distribution observed for *Anaplasma* spp. seroprevalence, we identified significant geographical distribution patterns for most of the other CVBPs, except for *B. burgdorferi* s.l., for which localised hotspots were detected (Figure 3). In particular, an east-to-west gradient was observed in *D. immitis* infections, with the highest infection prevalence occurring in the south-easternmost areas (Table 1, Figure 3). The municipalities from La Spezia province that showed a heightened probability of positive results for the infection demonstrated a mean prevalence of 14.0% (95% CI = 7.8–17.2). In the case of *R. conorii*, a west-to-east gradient trend was identified in the distribution of exposed dogs (*p* < 0.05), with the highest prevalence levels occurring in the south-westernmost regional limits (Table 1). In these areas, the prevalence of positivity against *R. conorii* antigen ranged from 9.90% to 29.6% at the municipal level, with an average seroprevalence of 28.7% (Table 1). Additionally, we identified several geographical hotspots for this species in central and eastern areas (Figure 3), although the odds of positive results in these areas were generally reduced (PR = 0.65 and PR = 0.82, respectively). Nevertheless, since 2021, there has been an increase in the number of positive results in specific localities within Genoa province (Figure 3), with prevalence ranging from 38.4% to up to 52.4%. With regard to *E. canis*, the highest prevalence levels were found to be clustered in central areas, with a range of 2.26 to 2.63%. In this case, a gradual decline in seroprevalence was noted towards both eastern and western areas (Table 1; Figure 3).

## 4. Discussion

This retrospective study contributes to characterise the epidemiology of several relevant CVBPs (*Anaplasma* spp., *Ehrlichia* spp., *R. conorii*, *B. burgdorferi* s.l., and *D. immitis*) in Liguria, a region for which data were scant. Exposure to at least one of the tested pathogens was detected in 18.8% of the 8584 tested dogs, while concomitant positivity for two or more pathogens was detected only in 1% of the dogs involved. Various retrospective studies have recently investigated the seroprevalence of CVBDs in other regions of Italy, including dogs from Sardinia [6], from Campania [26], and dogs sampled in Veterinary Teaching Hospitals in Central Italy [12,27]. The overall prevalence observed in Liguria is similar to that observed in dogs from southern Italy (19.6%), as determined by the SNAP^®^ 4DX^®^ PLUS test [26], and comparable to the result found by a veterinary blood bank in central Italy over a 10-year period of pre-donor screening (2012–2021) applying IFAT (25.71%) [27]. These results align with the overall prevalence of 25.6% found in Greece using rapid diagnostic tests [53]. It is evident that the overall positivity does not reflect the variations in the prevalence of individual pathogens across the various studies, which will be discussed below. In general, comparisons should be performed carefully due to the different diagnostic approaches applied. Indeed, the prevalence values recorded across studies varied considerably, depending on the dog populations analysed: for instance, very high seroprevalence values (72% for *R. conorii*, 46% for *E. canis*, 38% for *A. phagocytophilum*) were found in dogs from the Messina Strait (south Italy), with significantly higher values in public shelters than in private kennels, depending on tick management [25]. Several epidemiological studies have been conducted in other European countries, showing a wide variability in the prevalence of CVBDs. Such differences can be attributed to the biotic and abiotic variables specific to each region, as well as to the different diagnostic approach used [32,53,54].

Before proceeding to a further discussion of the results obtained, some limitations of the study should be pointed out. Firstly, no data on symptoms were available, which precludes any speculation on the effective impact of the investigated CVBDs on dogs’ health. Moreover, the data lacked information on the subjects’ sex, age, and breed, which prevented the inclusion of these variables in the statistical analysis. Finally, titration results were only available against the *R. conorii* antigen, and no further investigations, such as molecular analysis, were conducted on the positive samples. Therefore, except for *D. immitis*, it was not possible to identify the precise species implicated.

In this retrospective study, the highest seroprevalence (24.4%) was detected against the *R. conorii* antigen. Recent works have reported a seroprevalence for *Rickettsia* spp. ranging from 12.2 to 23.6% [6,12]. However, higher values were observed in southern Italy [24,25]. The high seroprevalence frequently detected in Italian dogs suggests a frequent exposure to *Rickettsia* spp., a persistent low-grade infection with a rickettsial organism, or organisms that cross-react with *R. conorii* antigens by IFA testing [55]. Serological diagnosis of rickettsial infections have been in use since the beginning of the last century, with IFA testing currently considered as the gold-standard serological assay [56]. However, cross reactions among the different species of *Rickettsia* spp. are known to occur [24,55,57] and have even been reported in the test’s instructions (https://www.agrolabo.it/en/product/fluo-rickettsia-conorii/ Accessed on 30 October 2024). Thus, the results of the IFAT test were not deemed specific for *R. conorii*, but rather indicative of exposure to SFG rickettsiae. Indeed, the presence of several species of SFG rickettsiae has been reported in a recent molecular survey on ticks and TBPs in wildlife from Liguria, which found 26.1% of the pools analysed to be positive for *Rickettsia* spp., and identified five species (*R. slovaca, R. monacensis, R. helvetica, R. massiliae*, and *R. raoultii*) [58]. Indeed, the occurrence of several tick species, such as *I. ricinus*, *R. sanguineus* s.l., *D. marginatus*, and *D. reticulatus*, acting as vectors of rickettsiae, was also reported to be widespread in the region [58]. The use of molecular tools has enabled the detection of several *Rickettsia* species in Italy over the past decade ([59] and cited references). Moreover, although *R. conorii*, the aetiological agent of Mediterranean Spotted Fever (MSF), was previously thought to be the only species responsible for human rickettsiosis in Italy, a number of other species, including *R. slovaca*, *R. monacensis*, *R. massiliae*, and *R. aeschlimannii*, have recently been identified as responsible for different forms of human disease [29,30,31]. Dogs have previously been used as epidemiological sentinels for human MSF [60], and proximity to seroreactive dogs has been identified as a risk factor for MSF in humans [61]. Furthermore, sub-clinically infected companion animals could serve as a reservoir for human tick-transmitted infectious agents [5].

As regards the remaining pathogen objects of this study, dogs’ exposure was detected (except for *D. immitis* when tested individually) through the use of the SNAP^®^ 4Dx^®^ Plus Test Kit, a commercially available in-clinic ELISA which has been reported to demonstrate high levels of sensitivity and specificity, both exceeding 90% overall [62]. Due to these good performances and the ease of administering the test, several large studies reporting the results of such rapid tests have been published lately [26,53,62], including a recent extensive retrospective study on results from 404,617 samples of dogs tested in 35 countries over a 5-year study period [32].

The rapid test used in this study detects both *A. phagocytophilum* and *A. platys*, though identifying the specific agent requires molecular tools. Accordingly, the positive results were here reported as positivity for *Anaplasma* spp. The seroprevalence of *Anaplasma* spp. in this study was 1.82%, which is comparable to findings in Central Italy (1.19%) [27] and Greece (2.3%) [53]. This seroprevalence value is lower than those reported in most of the studies available for Italy and Europe, where values typically range from 3% to 8%. For instance, seroprevalence values of 4.4% and 7.8% have been observed in Southern Italy [26,33]. In contrast, studies conducted in Central Italy have reported seroprevalences of 3.31, 4.7%, 8.76%, and 4.3% [12,18,63,64]. In Croatia and Hungary, seroprevalence levels of 4.5% [65] and 7.9% [66], respectively, have been recorded. Higher values (16.4%) were reported in dogs from Sardinia [6], and in symptomatic dogs from Southern Italy (39.8%) [4]. These findings indicate a widespread distribution of Anaplasmataceae bacteria throughout Italy and Europe. As previously noted, the reported seroprevalence of *Anaplasma* spp. is highly heterogeneous, mainly due to the variability of the diagnostic test used, the geographical context and the target dog population (e.g., symptomatic/non-symptomatic; stray/owned) assessed [4,26,27]. A strong molecular similarity has been described between human and canine isolates of *A. phagocytophilum* in Europe and the USA. The positive association between the distributions of human and canine cases in the USA highlights that dogs can serve as epidemiological indicators of potential human risk ([3] and cited references). Nonetheless, the low seropositivity observed over the years in this study, alongside the low PCR-positivity for *A. phagocytophilum* in ticks in Liguria [58], may suggest a relatively low risk for anaplasmosis in this region.

As regards *Ehrlichia* spp., the rapid tests used detect both *E. canis* and *E. ewingi*. However, the specific identification of these pathogens cannot be achieved without the use of molecular techniques. Consequently, the results for this pathogen were also reported as positivity for *Ehrlichia* spp., although *E. ewingi* has not been reported in Italy so far [23]. The total seroprevalence for *Ehrlichia* spp. found in this study (1.25%) was lower than the values recorded in several Italian studies, including 4.8% in Central Italy [12] and 9.9% in Sardinia [6]. Higher prevalence values (28.7–44.0%) have been reported in studies involving sick dogs [4,67]. Interestingly, the prevalence of this pathogen was higher in owned dogs in Liguria (6.5%) compared to shelter dogs (0.5%). This difference may be attributed to the fact that a significant proportion of the owned-dog population may be composed of hunting dogs, an at-risk animal category that is more susceptible to exposure to ticks during outdoor activities. In contrast, this exposure is likely to be limited for shelter dogs. Moreover, hunting dogs in the region are often kept in groups in private kennels that, in the absence of regular preventive treatments, are suitable habitats for *R. sanguineus* s.l., the most frequent vector of *Ehrlichia* spp. [20]. Similarly, a higher seroprevalence was also observed in owned dogs for *Rickettsia* spp. These differences could also be attributed to the differing exposure levels between the two dog populations, which might have influenced the higher positivity rates observed for *D. immitis* and *Rickettsia* spp. in inland dogs. Indeed, these mainly consisted of rural or hunting dogs, for which antiparasitic treatment might not be always accurate and regularly performed.

As regards exposure to *B. burgdorferi* s.l., we recorded the lowest prevalence levels in dogs compared to the exposure/infections observed for the other pathogens assessed. This finding agrees with the results obtained from dog populations in southern Italy [26]. The dog exposure to *B. burgdorferi* s.l. was sporadic and limited to specific localities within Liguria region. This pathogen has recently been found infecting a low proportion of engorged ticks collected from roe deer in the region [58]. However, previous studies revealed a moderate local circulation of several genospecies of *B. burgdorferi* in *I. ricinus* from the environment (18.2% of infection prevalence) [61]. Consequently, further research is necessary to better characterise the epidemiology of Lyme borreliosis in the region.

Additionally, a low prevalence of 0.84% was observed for *D. immitis*. It has to be noted that specific testing for *D. immitis* was usually requested in cases where symptoms were present, to confirm a positive blood examination conducted in clinic, or for dogs at risk, such as hunting dogs travelling to endemic areas. In fact, some of the dogs that tested positive for *D. immitis* were travelling from other regions with their owner of due to adoption (authors’ personal note). The low prevalence of the disease lends support to historical data, as the Liguria region was traditionally considered free from canine filariosis [68]. The prevalence of *D. immitis* reported herein is similar, although slightly higher than that recorded in a previous retrospective study conducted between 2004 and 2013 in the region, with 0.65% of 11,363 samples of canine sera testing positive for *D. immitis* antigens [68]. In Italy, the northern regions, especially those situated in proximity to the Po River valley, have historically constituted endemic areas [38]. However, recent findings indicate that the highest values are mainly concentrated in southern regions of the country [41]. At the European level, a generalised increase in cases has been observed since 2011, which has mainly been attributed to the expansion of the geographical range of its arthropod vectors (reviewed in [69]). Interestingly, a geographical pattern was observed in this study, with the eastern part of the region exhibiting a higher prevalence. This area is close to Tuscany, a region where *D. immitis* has been frequently reported since the 1970s [70]. As for the temporal pattern, the higher number of cases observed in autumn is consistent with mosquito-transmitted diseases, considering that the antigens are detectable not earlier than five to seven months post-infection [71]. As regards other temporal patterns, fluctuations in the number of tested animals showed an evident downward trend throughout the year, and 65.0% of tests conducted in the owned-dog population took place during the spring months. This pattern agrees with the current practice of testing for parasites such as *Leishmania infantum* and *D. immitis* in spring, at the start of the vector season.

Finally, the number of positive individuals followed an upward trend over time and within the years (*p* < 0.001) for all pathogens under study. This might be partly due to a higher performance of the diagnostic method, as over the years IDEXX has improved its point-of-care tests [72]. However, several factors have already been identified as the primary drivers behind the expansion of some disease vectors to date. For instance, the primary vector of Lyme borreliosis in Europe, the tick *I. ricinus*, has expanded its geographical limits towards more northern latitudes and higher altitudes across the continent, justified by changes in land use and wildlife management, among other factors [73,74,75]. *Aedes albopictus*, proven a competent vector of canine dirofilariasis, has also experienced a notable global expansion, largely attributable to human activities such as global trade and the use of public and private transportation [69]. Similar trends are suspected to be responsible for the recent expansion of *Ae. japonicus* and the emergence of *Ae. koreicus* in Europe [76]. In addition to the vectors, dynamics related to vertebrate hosts also play a role. Indeed, considering TBDs, the chance of being exposed to ticks in Liguria has grown lately, not only for the higher temperatures in winter months, but also for the increasing populations of wild hosts, such as wild ruminants and wild boars. Liguria territory is largely covered by mountains and hills, favouring the presence of wild animal species, with increasing populations of wild boars, wild ruminants, and wolves, often with a synanthropic attitude ([77] and cited references).

## 5. Conclusions

The study provides an up-to-date assessment of the exposure of the canine population to CVBDs in Liguria and aims to inform targeted interventions in this northwestern Italian region. Interestingly, a widespread exposure of dogs to *Rickettsia* spp. was observed, confirming a diffuse presence of this genus in the region. Moreover, an increasing number of positive cases throughout the years was observed for all pathogens, suggesting an increased exposure to pathogens transmitted by vectors. The increase in free-living wild ungulates and wild boar, in the investigated region as well as in Europe, together with anthropogenic and climatic changes, may increase the risk for CVBD transmissions and the public health risk in the case of zoonotic species.

## Figures and Tables

**Table 1:** Results of the analysis for the detection of circulating antigen of *Dirofilaria immitis*, and for circulating antibodies against *Rickettsia conorii, Ehrlichia* spp., *Anaplasma* spp., and *Borrelia burgdorferi*, with n of tested animals, number of positive animals, and total and provincial (Imperia, Savona, Genova, La Spezia) prevalences in dogs from Liguria, 2013–2023.

		Pathogen	No. of Tested Animals	No. of Positive Animals	Total Prevalence (%) [95% CI]	Imperia n. Pos (%)	Savona n. Pos (%)	Genoa n. Pos (%)	La Spezia n. Pos (%)
		*Dirofilaria immitis*	8368	70	0.84 [0.65–1.06]	9 (0.32)	20 (0.84)	12 (0.61)	29 (2.31)
Overall		*Anaplasma* spp.	5100	93	1.82 [1.47–2.23]	6 (0.26)	55 (4.51)	27 (4.70)	5 (0.48)
		*Borrelia burgdorferi* s.l.	5100	11	0.22 [0.11–0.39]	4 (0.18)	4 (0.33)	1 (0.17)	2 (0.19)
		*Ehrlichia* spp.	5100	64	1.25 [0.97–1.60]	10 (0.44)	32 (2.63)	13 (2.26)	9 (0.87)
		*Rickettsia conorii*	6024	1467	24.4 [23.3–25.5]	605 (28.7)	272 (23.7)	352 (20.1)	238 (23.6)
All			8584	1615	18.8 [18.0–19.7]	380 (19.2)	618 (21.5)	274 (21.5)	343 (14.0)
	Shelter dogs	*Dirofilaria immitis*	6229	47	1.08 [0.68–1.61]	9 (0.35)	5 (0.56)	11 (0.62)	22 (2.16)
		*Anaplasma* spp.	4472	73	1.63 [1.28–2.05]	5 (0.23)	39 (4.49)	24 (4.74)	5 (0.55)
		*Borrelia burgdorferi* s.l.	4472	8	0.18 [0.08–0.35]	4 (0.18)	3 (0.35)	0	1 (0.11)
		*Ehrlichia* spp.	4472	23	0.5 [0.3–0.77]	9 (0.41)	5 (0.58)	5 (1.00)	4 (0.44)
		*Rickettsia conorii*	5469	1288	23.6 [22.4–24.7]	590 (28.5)	172 (21.2)	333 (19.8)	193 (21.4)
	Owned dogs	*Dirofilaria immitis*	2139	23	1.08 [0.55–1.0]	0	15 (1.00)	1 (0.55)	7 (3.00)
		*Anaplasma* spp.	628	20	3.18 [1.96–4.88]	1 (1.41)	16 (4.57)	3 (4.35)	0
		*Borrelia burgdorferi* s.l.	628	3	0.48 [0.10–1.39]	0	1 (0.29)	1 (1.45)	1 (0.72)
		*Ehrlichia* spp.	628	41	6.53 [4.73–8.75]	1 (1.41)	27 (7.71)	8 (11.6)	5 (3.62)
		*Rickettsia conorii*	555	179	32.3 [28.4–36.3]	15 (35.7)	100 (29.9)	19 (26)	45 (42.5)

**Table 2:** Antibody titres against *Rickettsia conorii* antigen in dogs from the Liguria region, 2013–2023.

Antibody Titre Against *Rickettsia conorii* Antigen	Status of Infection	No. of Tested Animals (n = 6024)	%	Shelter Dogs n (%)	Owned Dogs n (%)
<1:80	Negative	4557	75.7	4181 (76.5)	376 (67.8)
≥1:80–1:160	Positive	982	16.3	865 (15.8)	117 (21.0)
1:320–1:640		410	6.8	358 (6.5)	52 (9.4)
≥1280		75	1.2	65 (1.2)	10 (1.8)

**Table 3:** Coinfections of canine vector-borne pathogens (CVBPs) occurring in the dog population of Liguria region between 2013 and 2023.

N of Coinfection	Pathogens	Total		Shelter Dogs		Owned Dogs	
		n	%	n	%	n	%
2	*Borrelia burgdorferi* s.l.—*Rickettsia conorii*	4	4.7	4	6.7	0	0.0
	*Ehrlichia canis*—*Rickettsia conorii*	20	23.5	8	13.3	12	48.0
	*Anaplasma* spp.—*Rickettsia conorii*	41	48.2	33	55.0	8	32.0
	*Anaplasma* spp.—*Borrelia burgdorferi* s.l.	2	2.4	1	1.7	1	4.0
	*Anaplasma* spp.—*Ehrlichia canis*	3	3.5	1	1.7	2	8.0
	*Dirofilaria immitis*—*Rickettsia conorii*	10	11.8	9	15.0	1	4.0
	*D. immitis*—*Ehrlichia canis*	1	1.2	1	1.7	0	0.0
3	*Anaplasma* spp.—*Ehrlichia canis*—*Rickettsia conorii*	3	3.5	2	3.3	1	4.0
4	*D. immitis*—*Anaplasma* spp.—*E. canis*—*R. conorii*	1	1.2	1	1.7	0	0.0

## Data Availability

The original contributions presented in this study are included in the article/Supplementary Materials. Further inquiries can be directed to the corresponding author.

## Supplementary Materials

The following supporting information can be downloaded at https://www.mdpi.com/article/10.3390/ani14233539/s1: Figure S1. Serological diagnostic activity conducted by the public veterinary diagnostic laboratories (*Istituto Zooprofilattico Sperimentale of Piemonte, Liguria and Valle d’Aosta*) on canine populations (owned and shelter dogs) in the Liguria region for the five canine vector-borne pathogens (CVBPs) under study from 2013 to 2023: (A) percentage of diagnostic activity performed on owned and shelter dogs by health service districts; (B) monthly trend in the total number of dogs tested, with a breakdown of owned and shelter dogs tested. Table S1. Composition of the sampled canine population by Ligurian provinces (NUTS2 level) and characteristics of the municipality of residence. Table S2. Optimal territorial, temporal, and environmental factors explaining the seroprevalence of canine vector-borne pathogens (CVBPs) in the dog population of the Liguria Region, 2013–2023.
