# Supplementary material for: Canine Vector-Borne Diseases (CVBDs) in Liguria, North-West Italy: A Retrospective Study over an 11-Year Period (2013–2023)
Source: Animals (Basel). 2024 Dec 7;14(23):3539. doi: 10.3390/ani14233539 (PMC11640262; doi:10.3390/ani14233539)
Supplement: Supplementary file 1 [file animals-14-03539-s001.zip › Table S1.pdf]

Table S1. Composition of the sampled canine population by Ligurian provinces (NUTS2 level) and characteristics of the municipality of residence.

	Owned dogs (n= 2228)	Shelter dogs (n= 6356)	Total (n; %)
**Geografical areas**			
Imperia	229	2651	2880 (33.6)
Savona	1551	895	2446 (28.5)
Genova	200	1784	1984 (23.1)
La Spezia	248	1026	1274 (14.8)
**Characteristics of municipality of residence**			
Urbanisation level			
Low	408	483	891 (10.4)
Moderate	1168	3738	4906 (57.1)
High	652	2135	2787 (32.5)
Altitude (in m a.s.l.)			
>250	1824	6011	7835 (91.3)
250 – 499	370	279	649 (7.5)
500 – 750	32	66	98 (1.2)
> 750	2	0	2 (0.02)
Geographical setting			
Inland	494	595	1089 (12.7)
Coastal	1734	5761	7495 (87.3)
