# Supplementary figures and images for: Canine Vector-Borne Diseases (CVBDs) in Liguria, North-West Italy: A Retrospective Study over an 11-Year Period (2013–2023)
Source: Animals (Basel). 2024 Dec 7;14(23):3539. doi: 10.3390/ani14233539 (PMC11640262; doi:10.3390/ani14233539)

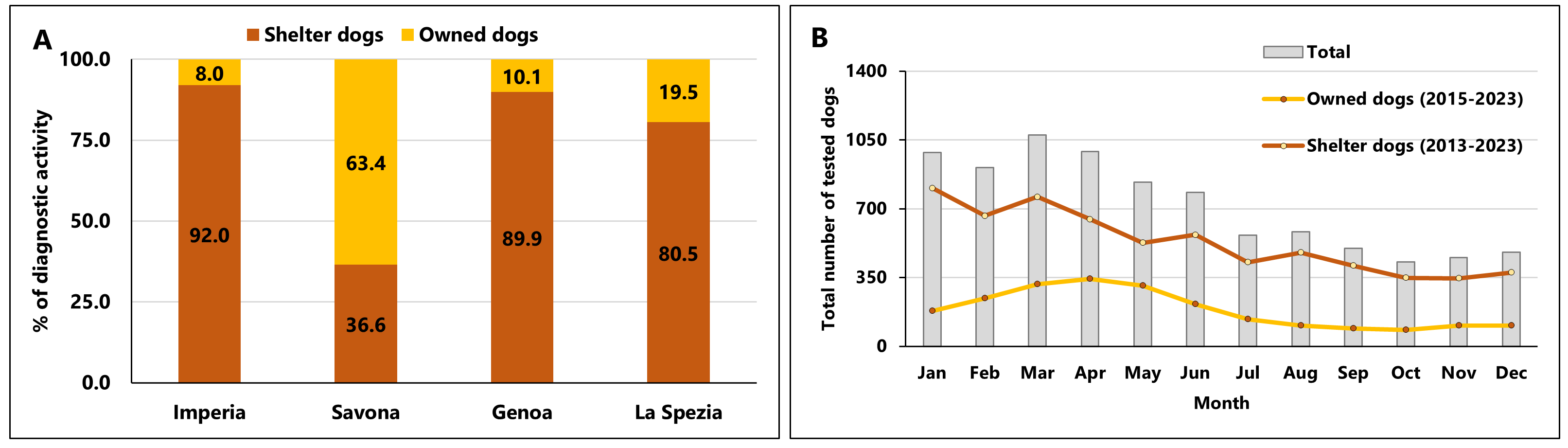

Supplement: Supplementary file 1 [file animals-14-03539-s001.zip › Figure S1A-B.tif]
